# Zinc thiazole enhances defense enzyme activities and increases pathogen resistance to *Ralstonia solanacearum* in peanut (*Arachis hypogaea*) under salt stress

**DOI:** 10.1371/journal.pone.0226951

**Published:** 2019-12-26

**Authors:** Suling Sang, Shaojian Li, Wanwan Fan, Na Wang, Meng Gao, Zhenyu Wang

**Affiliations:** Institute of Plant Protection, Henan Academy of Agricultural Sciences, Key Laboratory of Integrated Pest Management on Crops in Southern Region of North China; Henan Key Laboratory of Crop Pest Control; International Joint Research Laboratory for Crop Protection of Henan, Zhengzhou, China; Gyeongnam National University of Science and Technology, REPUBLIC OF KOREA

## Abstract

Crop plants always encounter multiple stresses in the natural environment. Here, the effects of the fungicide zinc thiazole (ZT) on propagation of *Ralstonia solanacearum*, a bacterial pathogen, were investigated in peanut seedlings under salt stress. Compared with water control, salt stress markedly reduced pathogen resistance in peanut seedlings. However, impaired pathogen resistance was alleviated by treatment with dimethylthiourea, a specific ROS scavenger, or ZT. Subsequently, salt stress or combined salt and pathogen treatment resulted in inhibition of photosynthesis, loss of chlorophyll and accumulation of thiobarbituric acid reactive substances, which could be reversed by ZT. In addition, ZT treatment suppressed the salt stress up-regulated Na^+^ content and Na^+^/K^+^ ratios in peanut roots. Furthermore, salt stress or combined salt and pathogen treatment impaired the activities of antioxidant (e.g. superoxide dismutase/SOD and catalase/CAT), and defense-related (e.g. phenylalanine ammonia lyase /PAL and polyphenol oxidase/PPO) enzymes, which could be rescued by addition of ZT. In contrast, only slight changes of SOD and CAT activities were observed in pathogen-infected seedlings. Similarly, activities of PAL and PPO were slightly modified by salt stress in peanut seedlings. These results suggest that the ZT-enhanced pathogen resistance can be partly attributed to the improvement of photosynthetic capacity and defense enzyme activities, and also the inhibition of Na^+^/K^+^ ratios, in this salt-stressed crop plant.

## Introduction

Plants are sessile and sensitive organisms that encounter a variety of environmental stresses, including drought, high salinity, heavy metal, cold stresses and pathogen attack, throughout their life cycle. Salinity is a major environmental factor limiting plant photosynthesis and crop yields, which is due to the low osmotic potential of soil solution-specific ion effects, nutritional imbalance or a combination of these factors [1,2]. Moreover, salinity causes a disturbance of the K^+^/Na^+^ ratio [2]. Salt stress also leads to oxidative stress through an increase in formation of reactive oxygen species (ROS). To mitigate the oxidative damage initiated by ROS, plants have developed a complex antioxidant defense system, including low molecular mass antioxidants as well as antioxidant enzymes, such as superoxide dismutase (SOD) and catalase (CAT) [3,4].

*Ralstonia solanacearum* (previously named *Pseudomonas solanacearum*) can cause bacterial wilt, one of the most important bacterial diseases worldwide [5]. Many tropical and sub-tropical crop species (e.g. peanut, tomato, tobaaco and pepper) would suffer from this soil-borne, vascular pathogen [6]. It can induce systemic resistance in some plants, such as increasing the activities of plant defense-related enzymes phenylalanine ammonia lyase (PAL) and polyphenol oxidase (PPO) [7,8]. PAL is a key enzyme in the phenylpropanoid biosynthetic pathway, and also plays an important role in flavonoid production and lignin biosynthesis [7]. PPO can catalyze the formation of lignin and other oxidative phenols, thereby contributing to the formation of defense barriers by reinforcing the cell structure, to protect against pathogens [8].

Farmers and breeders have long known that often it is the simultaneous occurrence of several abiotic stresses, rather than a particular stress condition, that is most lethal to crops [9]. Recent evidence shows that plants respond to multiple stresses in a manner different from how they do to individual stresses [10–12]. Rather than being additive, the presence of an abiotic stress can have the effect of reducing or enhancing susceptibility to a biotic pest or pathogen, and vice versa [13]. For example, Wiese et al. [14] reported that salt treatment can impart increased resistance of the host plant to the biotrophic pathogen *Blumeria graminis f*.*sp*. *hordei*. Based on the “water-model” hypothesis, water is required for pathogen development in plant-pathogen interactions [15]. In accordance with this hypothesis, enhanced pathogen resistance has been observed in host plants under drought conditions [16].

Agrochemicals (including pesticides) have always been used by farmers for controlling pathogen development and pest attack in crop plants [17]. For example, zinc thiazole (ZT) is a highly efficient and commonly used pesticide for controlling many kinds of crop diseases [18,19]. However, pesticide application also poses a severe risk to environmental pollution to damage the ecosystem, including plants, water and soil [20].

Compared with single stress, there is little data to show how plant resistance can be improved under multiple stress conditions. Here, we addressed several questions. Firstly, we tested whether salt stress can enhance or reduce bacterial pathogen resistance in peanut plants. Secondly, we investigated whether salt stress affecting the pathogen response could be modified by ZT. Thirdly, we explored what the possible mechanisms underlying this phenomenon could be. This is the first report describing the effect of a pesticide on pathogen resistance in plants under abiotic stress.

## Results

### Effects of salt stress on pathogen development

**Fig 1:**
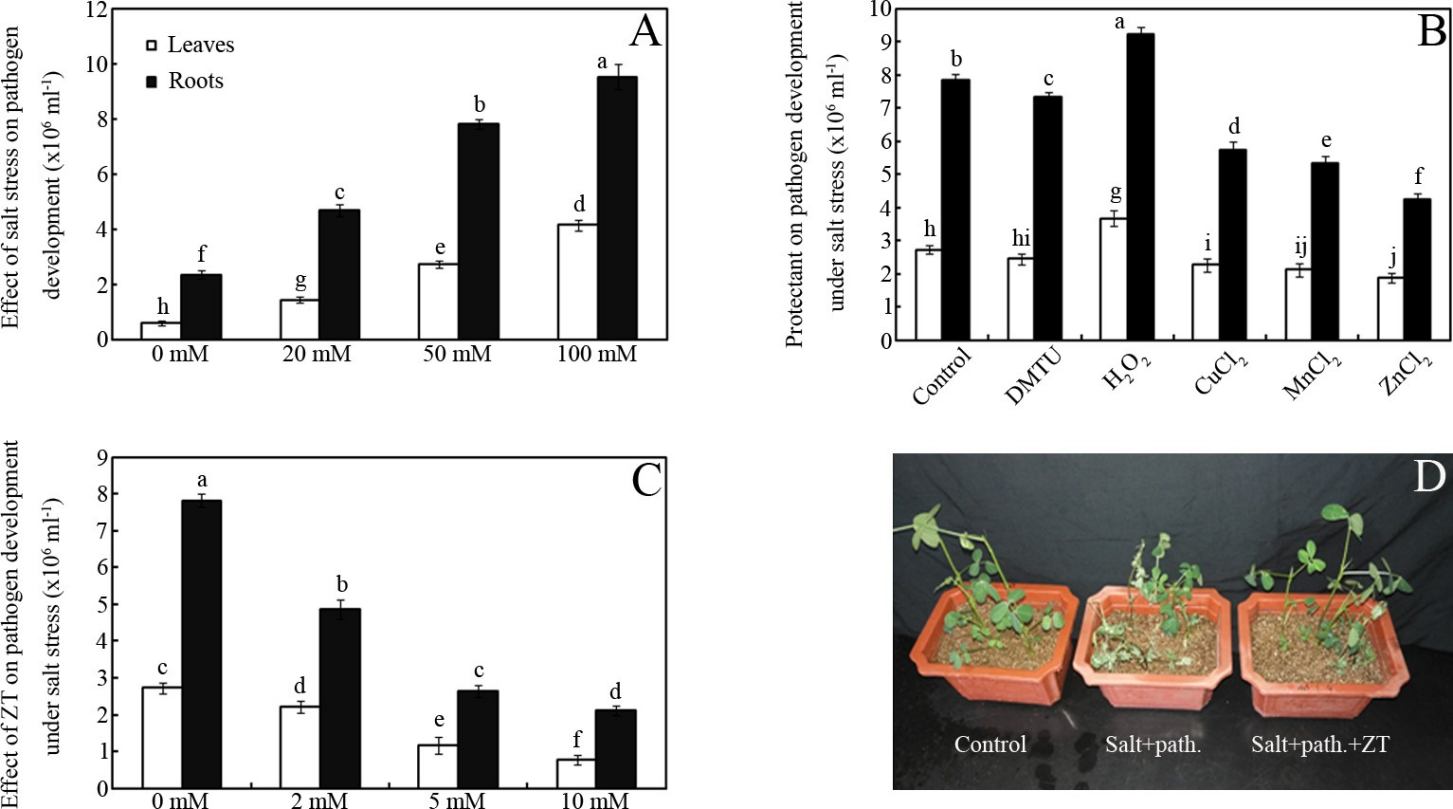
Effects of salt stress on pathogen development. Effects of different concentrations of NaCl (A), 50 mM NaCl + reagent (B) and 50 mM NaCl + ZT (C,D) on pathogen development on peanut seedlings. Bars represent standard deviations of means (*n* = 5); means indicated by the same letter are not significantly different (Duncan’s multiple range test, *P* < 0.05) among treatments.

Salt stress markedly impaired bacterial wilt resistance in peanut seedlings. As shown in Fig 1A, bacterial numbers increased approximately 1.8-, 3.7- and 6.1-fold in 20, 50 and 100 mM NaCl-treated peanut leaves, respectively, compared to those of the controls (salt free group) after inoculation for seven days (*p* < 0.05). Similar change patterns were also monitored in roots (Fig 1A). In addition, 50 mM NaCl was used in all further experiments.

However, salt stress-impaired pathogen resistance was significantly, but slightly modified by dimethylthiourea (DMTU), a specific ROS scavenger (Fig 1B). For example, approximately 9% and 7% reduction in bacterial numbers was monitored in DMTU-treated seedling leaves and roots, respectively, compared to controls, after inoculation for seven days (Fig 1B; *p* < 0.05). In contrast, H_2_O_2_ addition significantly enhanced salt stress-induced pathogen development (Fig 1B). For example, H_2_O_2_ caused an increase of approximately 34% and 18% in bacterial numbers in leaves and roots of salt-stressed seedlings, respectively, after inoculation for seven days (Fig 1B; *p* < 0.05). However, Cu^2+^, Mn^2+^ and Zn^2+^ treatments significantly arrested pathogen development. For example, Cu^2+^, Mn^2+^ and Zn^2+^ reduced bacterial numbers by 18%, 23% and 31% in salt-stressed seedling leaves, respectively, compared to controls, after inoculation for seven days (Fig 1B; *p* < 0.05). In addition, similar change patterns were also monitored in roots (Fig 1B; *p* < 0.05).

Salt stress-impaired pathogen resistance could be partly reversed by ZT in peanut seedlings. For example, treatment with 2, 5 and 10 mM ZT reduced bacterial numbers by approximately 19%, 57% and 72%, respectively, in leaves of salt-stressed (50 mM NaCl) seedlings after inoculation for seven days (Fig 1C; *p* < 0.05). In addition, similar change patterns were also monitored in roots (Fig 1C; *p* < 0.05). However, the combined salt and pathogen-induced wilting symptoms in mature soil-grown plants could be mitigated by ZT after 15 days (Fig 1D; *p* < 0.05).

### Effects of ZT on leaf photosynthesis

Salt stress-impaired leaf photosynthesis (net photosynthesis rate, P_N_ and stomatal conductance, g_s_) was also significantly alleviated by ZT. For example, salt stress decreased net photosynthesis by approximately 28% and stomatal conductance by 30%, compared to those of controls (Table 1; *p* < 0.05). However, 5 mM ZT treatment caused an approximate 28% increase in net photosynthetic rates and 34% in stomatal conductance, in salt-stressed seedlings (Table 1; *p* < 0.05). In addition, similar changes in patterns were also observed in the salt-stressed seedlings after pathogen inoculation. For example, 5 mM ZT treatment increased net photosynthesis by approximately 35% and stomatal conductance by 30% in salt-stressed seedlings after pathogen inoculation for seven days (Table 1; *p* < 0.05).

**Table 1:** Effects of ZT on net photosynthetic rates and stomatal conductance.

	Water	Salt stress	Pathogen	Salt + pathogen
P_N_ (- ZT): μmol CO_2_ m^-2^ s^-1^	17.2 ± 0.2^a^	12.3 ± 0.3^a^	15.2 ± 0.1^a^	11.3 ± 0.4^a^
P_N_ (+ ZT): μmol CO_2_ m^-2^ s^-1^	18.7 ± 0.3^b^	15.8 ± 0.2^b^	16.2 ± 0.2^b^	15.2 ± 0.1^b^
g_s_ (- ZT): mmol m^-2^ s^-1^	115.4 ± 2.3^A^	80.3 ± 1.9^A^	101.4 ± 2.7^A^	75.9 ± 2.4^A^
g_s_ (+ ZT): mmol m^-2^ s^-1^	122.1 ± 1.8^B^	107.3 ± 3.5^B^	108.4 ± 1.4^B^	98.9 ± 3.2^B^

ZT, zinc thiazole; P_N_, net photosynthesis rate; g_s_, stomatal conductance

In each row, the values represented by the same lower case and capital letter in P_N_ and g_s_, respectively, are not significantly different at *p* < 0.05 (Duncan’s multiple range test).

### Effects of ZT on chlorophyll and TBARS content

**Fig 2:**
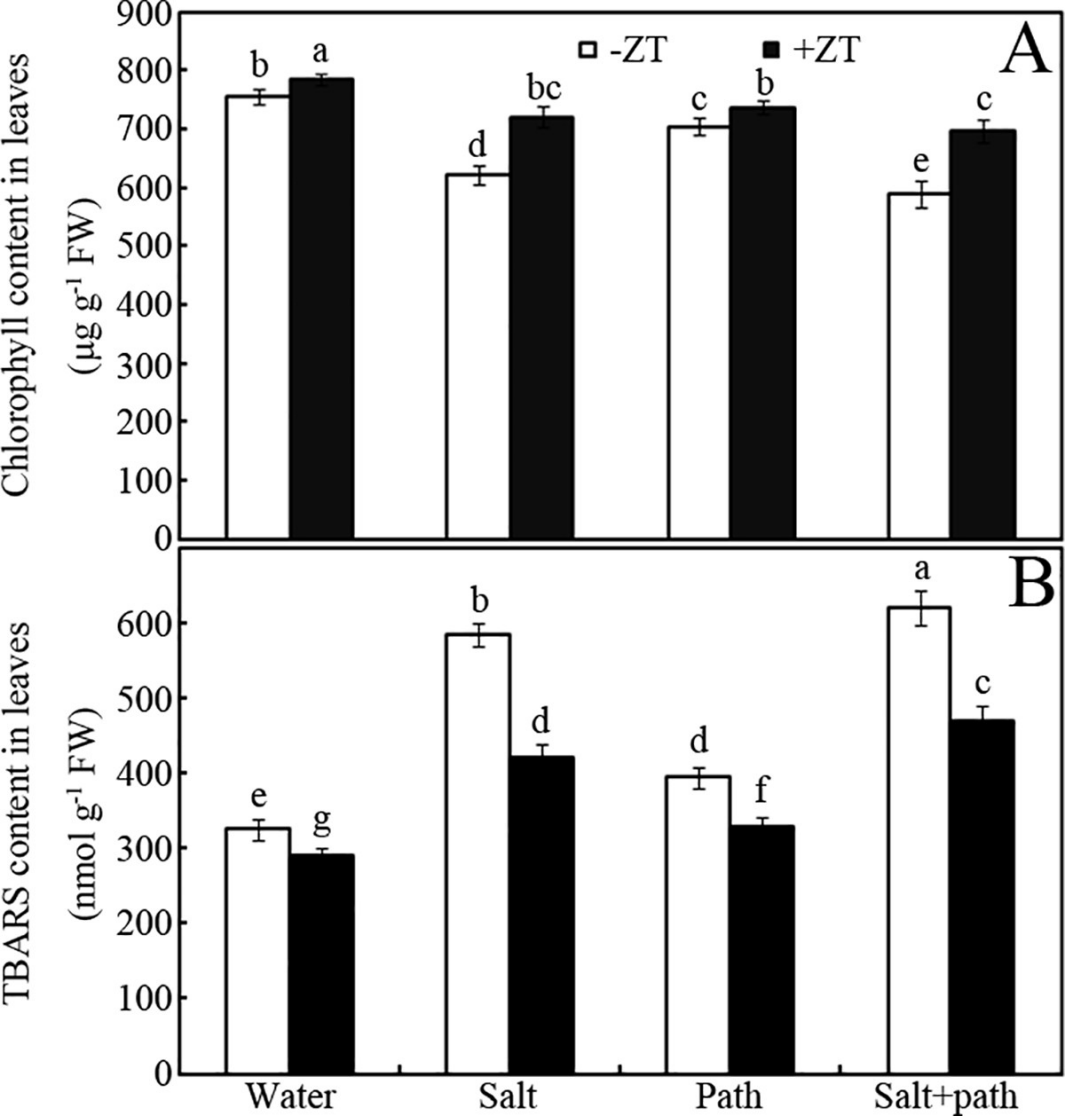
Effect of ZT on chlorophyll and TBARS content. Effect of 5 mM ZT on chlorophyll (A) and TBARS (B) content of peanut leaves after inoculation for seven days under salt stress (50 mM NaCl). Bars represent standard deviations of means (*n* = 5); means followed by the same letter are not significantly different (Duncan’s multiple range test, *P* < 0.05) amongst treatments.

Impairment of chlorophyll levels by salt stress could be reversed by ZT in seedlings after inoculation with bacteria for seven days (Fig 2A). For example, 5 mM ZT treatment increased chlorophyll levels by approximately 4%, 16%, 5% and 18% in peanut leaves in water, salt, pathogen and salt + pathogen treatments, respectively, compared to that of the control (-ZT) (Fig 2A; *p* < 0.05). Accordingly, salt stress-enhanced thiobarbituric acid reactive substances (TBARS) levels were down-regulated by ZT in seedlings after inoculation for seven days (Fig 2B). For example, 5 mM ZT decreased TBARS content in peanut leaves by approximately 11%, 28%, 16% and 24% in water, salt, pathogen and salt + pathogen treatments, respectively, compared to that of the control (-ZT) (Fig 2B; *p* < 0.05).

### Effects of ZT on Na^+^/K^+^ ratios

**Fig 3:**
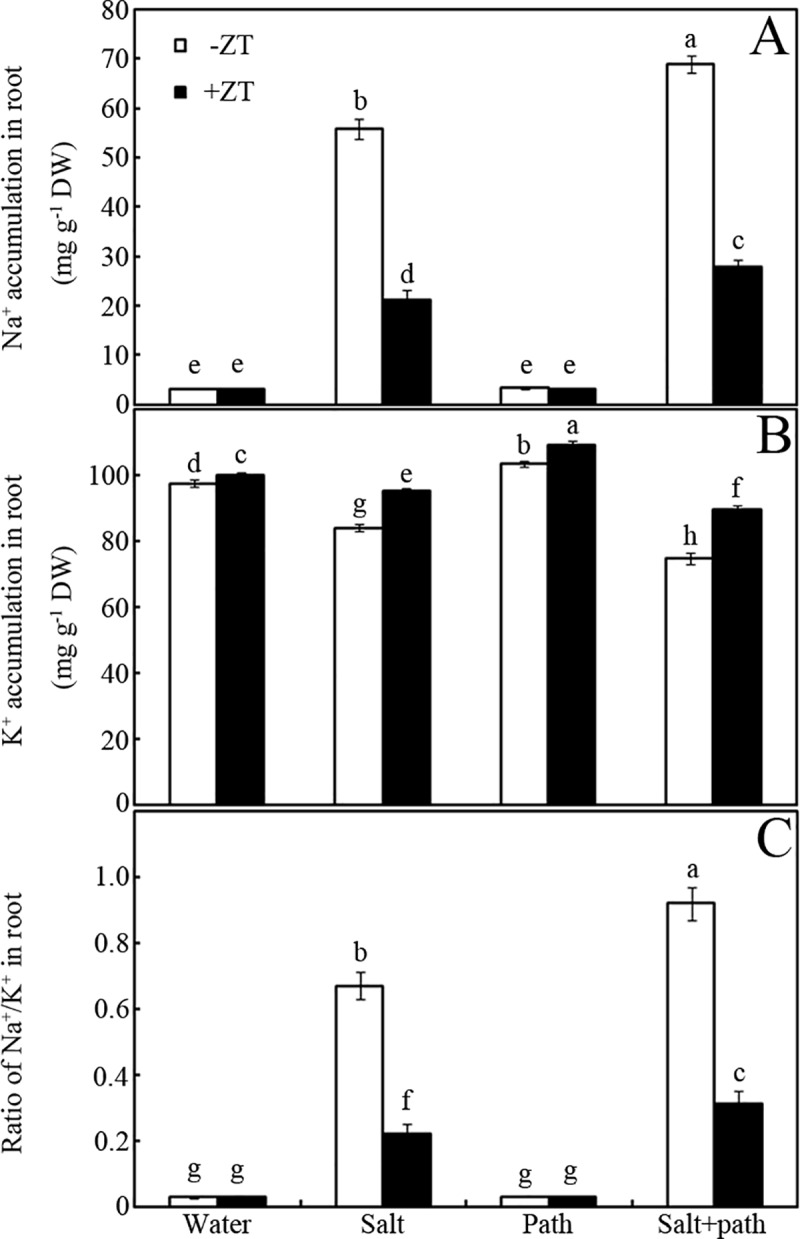
Effects of ZT on Na^+^/K^+^ ratios. Effects of 5 mM ZT on content of Na^+^ (A), K^+^ (B) and Na^+^/K^+^ ratios (C) of peanut roots after inoculation for seven days under salt stress (50 mM NaCl). Bars represent standard deviations of means (*n* = 5); means followed by the same letter are not significantly different (Duncan’s multiple range test, *P* < 0.05) amongst treatments.

Na^+^ and K^+^ content and Na^+^/K^+^ ratios were significantly modified by salt stress (Fig 3). For example, salt stress increased Na^+^ content by 17-fold, and Na^+^/K^+^ ratio by 22-fold, but decreased K^+^ content by 14% over the control (-ZT) (Fig 3). However, this salt stress-increased Na^+^ content and Na^+^/K^+^ levels in peanut seedlings could be significantly reversed by ZT addition (Fig 3A and 3C). As shown in Fig 3A, ZT treatment reduced Na^+^ content by approximately 62% and 60% in salt or salt + pathogen-treated seedling roots, respectively, over the controls (*p* < 0.05). Accordingly, treatment with ZT decreased Na^+^/K^+^ ratios by approximately 67% and 64% in salt or salt + pathogen-treated seedling roots, respectively, over the controls (-ZT) (Fig 3C; *p* < 0.05). In addition, ZT significantly improved the K^+^ levels in peanut seedling roots under salt stress (Fig 3B). For example, a 13% (salt group) and 19% (salt+pathogen group) increase in K^+^ content was observed in ZT-treated seedling roots compared to controls (-ZT) (Fig 3B; *p* < 0.05).

### Effects of ZT on antioxidant enzyme activities

**Fig 4:**
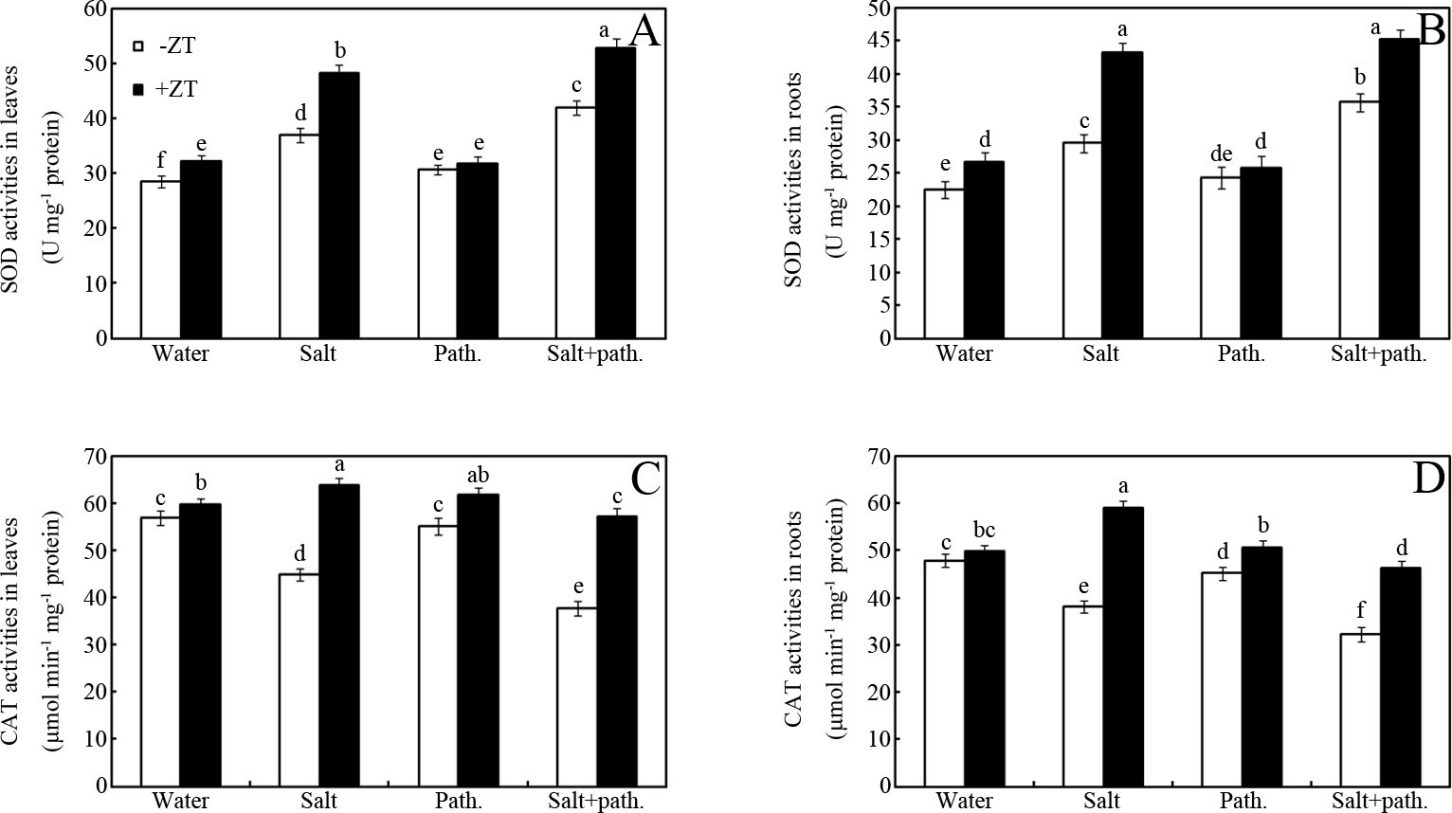
Effects of ZT on antioxidant enzyme activities. Effects of 5 mM ZT on SOD (A, B) and CAT (C, D) enzyme activities of peanut leaves and roots after inoculation with bacteria for seven days under salt stress (50 mM NaCl). Bars represent standard deviations of means (*n* = 5); means followed by the same letter are not significantly different (Duncan’s multiple range test, *P* < 0.05) amongst treatments. SOD, superoxide dismutase; CAT, catalase.

Compared with water control, salt stress and pathogen inoculation increased SOD and CAT activities to different extents (Fig 4). For example, increases in SOD activity of approximately 29%, 7.3% and 46.5% were measured in salt stress, pathogen and salt + pathogen-treated seedling leaves, compared to those of the water treatment group (Fig 4A; *p* < 0.05). Similar changes in patterns were also monitored for SOD in roots, and CAT enzymes in leaves and roots (Fig 4B–4D). Interestingly, ZT increased SOD and CAT activities under salt stress, pathogen challenge or combined stress conditions (Fig 4). For example, ZT increased CAT activity by approximately 5%, 42%, 12% and 51% in water, salt, pathogen and salt + pathogen treatments, respectively, in leaves of peanut seedlings over the control (-ZT) (Fig 4C; *p* < 0.05). Similar changes in patterns were also monitored for SOD (Fig 4A and 4B).

### Effects of ZT on pathogen defense-related enzyme activities

**Fig 5:**
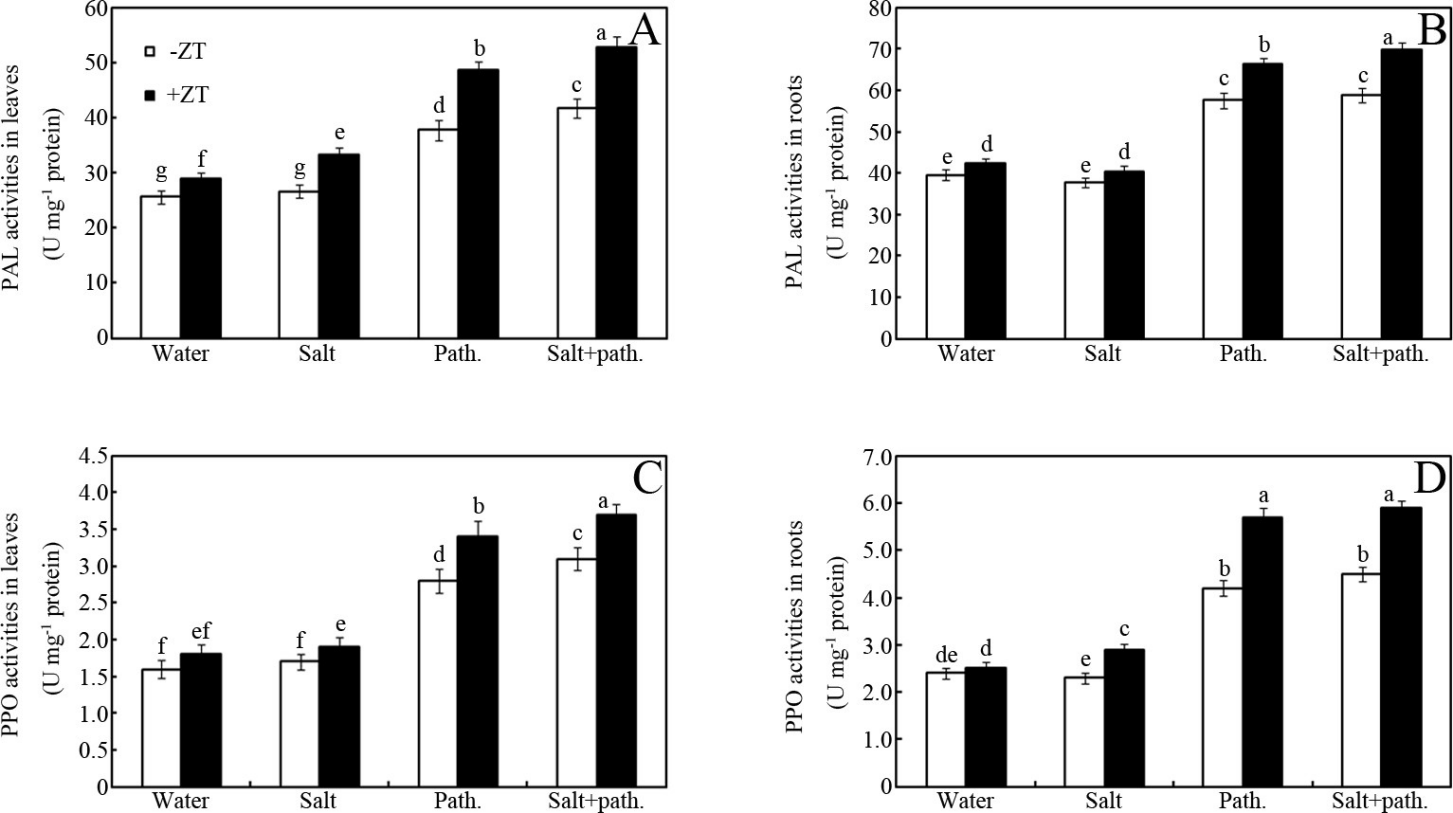
Effects of ZT on pathogen defense-related enzyme activities. Effects of 5 mM ZT on PAL (A, B) and PPO (C, D) of peanut leaves and roots after inoculation for seven days under salt stress (50 mM NaCl). Bars represent standard deviations of means (*n* = 5); means followed by the same letter are not significantly different (Duncan’s multiple range test, *P* < 0.05) amongst treatments. PAL, Phenylalanine ammonia-lyase; PPO, Polyphenol oxidase.

Compared with salt stress, pathogen challenge significantly induced PAL and PPO activities in peanut seedlings (Fig 5). For example, approximately 6%, 47% and 63% increase in PAL activity was measured in salt stress, pathogen challenge and salt + pathogen treatments, respectively, compared to that of the water treatment in seedling leaves (Fig 5A; *p* < 0.05). Similar changes in patterns were also observed in PAL and PPO enzymes in seedling leaves and roots (Fig 5B–5D). Interestingly, ZT treatment also further enhanced the activities of these enzymes (Fig 5). For example, ZT increased activity of PAL by approximately 7%, 9%, 15% and 20% in water, salt stress, pathogen and salt + pathogen treatments, respectively, in seedling roots over the control (-ZT) (Fig 5B; *p* < 0.05). Similar changes in patterns were also observed in PAL and PPO enzymes in leaves and roots (Fig 5).

## Discussion

The “water model” hypothesis considers that water is required for pathogen development in the infected site [15]. Alongside drought, salt treatment also induces water deficit in plants, and theoretically, increased bacterial wilt resistance should be detected with salt stress. However, in this study, impaired pathogen resistance was observed during *Ralstonia*-peanut interaction under salt stress conditions, with increased bacterial growth (Fig 1A). Thus, this hypothesis does not seem to be valid for illustrating a reduction in bacterial growth in peanut seedlings under salt stress.

It has been suggested that effective defense against biotrophic pathogens is largely due to programmed cell death in the host, and associated activation of defense responses regulated by the salicylic acid-dependent pathway [21]. In contrast, necrotrophic pathogens benefit from host cell death, so they are not limited by cell death and salicylic acid–dependent defenses [21]. *Ralstonia solanacearum* (RS) can propagate in peanut plants via a necrotrophic lifestyle and cause peanut bacterial wilt [22]. Salt stress can induce ROS over-production, which can lead to oxidative stress and subject plant cells to a more fragile state [4]. Thus, we hypothesized that salt stress-mediated ROS production may promote this pathogen development in peanuts. To test this hypothesis, antioxidants (such as DMTU) and oxidants (such as H_2_O_2_) were applied to salt-stressed seedlings after pathogen inoculation (Fig 1B). Results showed that antioxidants such as DMTU can partly arrest pathogen development in peanut seedlings (Fig 1B). Accordingly, H_2_O_2_ further aggravated disease development (Fig 1B). One plausible explanation for this phenomenon can be attributed to the salt stress-mediated ROS production which would impair plant cell function, thereby improving the necrotrophic lifestyle choice for the bacteria.

Previous data has shown that certain heavy metals can defend plants against biotic stress [23,24]. Here, three heavy metals (Cu^2+^, Mn^2+^ and Zn^2+^) were used for evaluation of their possible roles during this plant-pathogen interaction. Results showed that all these heavy metals, especially Zn^2+^, could markedly arrest bacterial wilt development to different extents (20–30%) in peanut seedlings (Fig 1B). However, this slightly protective effect of a single heavy metal cannot address the agricultural demand for a suitable agent to control the disease. Thus, the agrochemical ZT, a Zn-containing pesticide that has been used under favorable conditions [18,19], was chosen. We then investigated whether this pesticide could enhance bacterial wilt resistance for peanut seedlings under salt stress.

As shown in Fig 1C, salt stress-impaired pathogen resistance could be rescued by ZT. It aroused an interesting problem as to why low levels of ZT (e.g. 2 mM) could improve pathogen resistance as well as 10 mM ZnCl_2_ under salt stress. It has been shown that enhancement of primary metabolism (e.g. photosynthesis) can enhance pathogen resistance in plants [25,26]. Thus, one plausible explanation may be attributed to the roles of ZT in regulating photosynthesis under salt stress conditions (Fig 1D).

To test this speculation, the effect of ZT on photosynthesis was measured (Table 1). Data showed that salt stress-impaired photosynthetic function (e.g. P_N_ and g_s_) could be significantly recovered by ZT. We therefore showed that ZT can improve peanut seedling growth via regulating its photosynthesis. In a further experiment, chlorophyll levels and TBARS accumulation were monitored in salt-stressed peanut plants after pathogen inoculation (Fig 2). Results showed that salt stress impaired chlorophyll levels could be significantly restored by ZT (Fig 2A). This is in accordance with ZT-improved photosynthetic capacity in peanut seedlings under salt stress (Table 1). Additionally, salt stress-induced TBARS accumulation could be partially reversed by ZT for peanut seedling leaves (Fig 2B). It is well known that both Zn and the organic group of ZT have bactericidal effects [23]. However, Zn as a nutrient element also can favor plant growth (e.g. photosynthesis) under salt stress conditions. In fact, this improved primary metabolism would benefit the biosynthesis of more defense compounds. Thus, our results suggest that ZT-enhanced pathogen resistance can be partly attributed to its improving primary metabolism (e.g. photosynthesis) under salt stress.

In addition, photosynthetic capacity is also closely associated with Na^+^ and K^+^ assimilation and partitioning in salt-stressed plants [27]. Thus, accumulation levels of Na^+^ and K^+^, coupled with Na^+^/K^+^ ratios, were also measured for evaluating the salt stress tolerance mechanisms of peanut seedlings (Fig 3). Results showed that ZT significantly suppressed Na^+^ accumulation and enhanced K^+^ assimilation, coupled with down-regulation of the Na^+^/K^+^ ratios, in salt-stressed peanut seedlings (Fig 3). Our data showed that ZT treatment can maintain the ion homeostasis of peanut seedlings under salt stress. One plausible explanation can be attributed to the antagonistic effects of Zn^2+^ against Na^+^. However, the direct defense mechanisms by which ZT improves pathogen resistance under salt stress are still elusive. In addition, previous data show that Zn is an indispensable element of the SOD enzyme [28] or activator of defense enzymes [29]. Thus, the activities of antioxidant (e.g. SOD and CAT) and defense enzymes (e.g. PAL and PPO) were measured in this study (Figs 4 and 5).

Results showed that ZT significantly increased SOD and CAT activities in salt-stressed seedlings, irrespective of pathogen infection (Fig 4). Previous data showed that SOD and CAT are two key antioxidant enzymes for abiotic stress tolerance in plants [4]. Our results suggest that ZT-enhanced photosynthetic capacity can therefore be partly attributed to the improved antioxidant enzyme activities under salt stress conditions.

In further experiments, the effects of ZT on pathogen-related defense enzyme activities were also measured (Fig 5). Here, activities of PPO and PAL were also up-regulated by ZT in the salt-stressed seedlings after pathogen inoculation (Fig 5). Previous data also showed that Zn element can enhance PAL and PPO enzyme activities in *Rhizoctonia* infected clusterbean seedlings [29]. Our data suggest that ZT-enhanced pathogen resistance in salt-stressed peanuts can be partly attributed to its up-regulation of defense enzyme (e.g. PAL and PPO) activities, probably via zinc action.

Compared with ZnCl_2_, the Zn-containing pesticide ZT exhibited greater inhibitory effects for pathogen development in peanut seedlings under salt stress (Fig 1B and 1C). This suggests that the thiazole group of ZT also plays a key role in arresting pathogen development and alleviating salt toxicity in peanut seedlings. However, the underlying mechanisms of thiazole in protecting peanut seedlings under these combined stresses are still elusive [30,31]. Thus, more experimental data are required to test this hypothesis.

Based on the experimental data mentioned above, we can attribute the ZT-enhanced pathogen resistance in salt-stressed peanut plants to the factors below: Zn as a nutrient can improve plant growth and primary metabolism, which can improve the biosynthesis of more antioxidants (e.g. SOD and CAT) and defense compounds (e.g. PPO and PAL) under salt stress; the direct bactericidal effects of both Zn and the thiazole group of ZT; and this alleviated pathogen infection would also further improve plant salt tolerance and vice versa.

In summary, some interesting conclusions can be drawn here. Firstly, salt stress can profoundly reduce bacterial wilt resistance for peanut seedlings. Secondly, ZT addition can significantly improve pathogen resistance for salt-stressed peanut seedlings. Thirdly, salt stress increases Na^+^/K^+^ ratios and impairs leaf photosynthesis, which can be reversed by ZT in peanut seedlings after inoculation. Fourthly, ZT can increase activities of SOD, CAT, PPO and PAL in pathogen-infected peanut seedlings under salt stress. This is the first report of ZT in regulating pathogen development in host plants under abiotic stress. ZT is cheap and readily available, making it suitable for widespread application in agriculture to improve bacterial wilt resistance in salt-stressed peanut plants.

## Materials and methods

### Bacterial strain preparation

The *R*. *solanacearum* strain GMI1000 is a standard strain obtained from College of Plant Protection, Huazhong Agricultural University [32]. The strain was grown at 28°C in B broth (containing 10 g L^-1^ Difco Bacto-Peptone; 1 g L^-1^ Difco Yeast Extract; 1 g L^-1^ Difco Casamino acids) or BGT solid medium (containing 15 g L^-1^ Bacto-Agar, 5 g L^-1^ glucose and 50 mg L^-1^ triphenyltetrazolium chloride) [33], and stored at –70°C for long-term storage. Before inoculation, the stock culture was thawed and streaked on a selective agar medium containing 200 μg ml^-1^ streptomycin sulphate. After inoculation at 28°C for 48 h, bacteria were collected using a plastic spatula and suspended in distilled water. The suspension was adjusted to 5 ✕ 10^8^ CFU ml^-1^ by means of a spectrophotometer (OD_660_ = 0.24) and diluted with water to give a final concentration of 10^7^ CFU ml^-1^.

### Bacterial inoculation and development assay

Peanut genotype J62 was provided by Oil Crop Research Institute, Chinese Academy of Agricultural Sciences [34]. The seeds were sterilized for 20 min with a 12% sodium hypochlorite solution, washed several times with sterile water and sown on Murashige and Skoog medium [35]. Plantlets grown for eight days at 20°C in a growth chamber (16 h: 8 h light/dark cycle, 200 μmol photon m^-2^ s^-1^, 70% relative humidity) were then transferred to plastic boxes (containing fine sand, watered with 1:10 Hoagland solution) and grown for two weeks in short day conditions (10 h/14 h light/dark) under constant light at 500 μE m^–2^ s^–1^. Root inoculations were performed according to the following protocol: approximately 0.5 cm was cut from the root end and the wounded roots of the peanut plants were immersed for 3 min in a 100 ml suspension containing 10^7^~10^8^ bacteria per ml. The plants were then transferred to a growth chamber at 25°C (16 h/8 h light/dark, constant light at 500 μE m^–2^ s^–1^) until symptoms appeared. At zero and seven days post-infection (dpi), pathogen-infected tissues (leaves and roots) were collected, and the bacterial populations in the infected tissues were monitored as described [8]. Leaves and roots were harvested and sterilized with 70% ethanol for 1 min, rinsed three times in sterile water, patted dry, weighed, crushed, and resuspended in water. Bacterial concentrations were determined by dilution plating on SMSA medium. At least five independent experiments were performed for the disease resistance assay. In addition, all work was performed in Peanut Disease Laboratory in Henan Academy of Agricultural Sciences.

### Seedling treatment

Ten-day-old seedlings of uniform size were selected to determine the concentration of ZT that would effectively protect them against infection. The seedlings were sprayed with 0, 2, 5 and 10 mM concentrations of ZT solution. Each treatment group contained five replicates of ten seedlings. After three days of pre-cultivation, each replicate group was randomly divided into several treatments: water, salt, pathogen, salt + pathogen. For treated seedlings, the concentration of the reagent (including NaCl, H_2_O_2_, DMTU and heavy metals) used is described in the text. A misting system was used to maintain relative humidity above 70%. Based on preliminary results, we selected 5 mM ZT for further studies with 100 uniformly-sized seedlings.

### Chlorophyll content assay

Chlorophyll extraction and content assay was performed according to the method of Lichtenthaler [36]. Leaf sections of approximately the same areas were taken from peanut leaves, ground to a powder, and extracted in 5 ml of 80% acetone. After centrifugation at 3,000 x *g* for 5 min under 4°C conditions, total chlorophyll content was determined at 663 nm, 652 nm and 645 nm.

### Lipid peroxidation assay

Lipid peroxidation was determined by thiobarbituric acid reactive substances (TBARS) accumulation [37], where leaf tissues were homogenized in 10% trichloroacetic acid (TCA) and centrifuged at 4,000 ✕ *g* for 10 min. The supernatant was mixed with 0.6% thiobarbituric acid (TBA) and heated at 95°C. Following this, it was quickly cooled and centrifuged again. The supernatant was analyzed using UV-Vis spectroscopy (Shimadzu, Kyoto, Japan) at 532 nm and 600 nm to determine TBARS concentration, based on an extinction coefficient of 0.155 M^-1^ cm^-1^.

### Na^+^/K^+^ ratio assay

Whole seedlings were used to determine the contents of Na^+^ and K^+^ by using an inductively coupled plasma–atomic emission spectrometer (ICP-OES, Perkin-Elmer Optima 2100DV, USA). The seedlings were rinsed with deionized water three times, killed at 110°C for 10 min, dried at 70°C for 48 h, and incinerated at 550°C for 6 h. An aliquot of sample ash was dissolved in 0.5 M HCl solution. Concentrations of Na^+^ and K^+^ in the solution were measured.

### Leaf photosynthesis assay

Net photosynthetic rate (P_N_) and stomatal conductance to water vapor (g_s_) were measured in peanut seedlings using a LCA-4 portable photosynthesis system (Analytical Development, Kings Lynn, UK). The photosynthetic photon flux density was maintained at 900 mmol photons m^-2^ s^-1^ on the cuvette surface by a portable light unit model PLU2-002 (Analytical Development, UK). All measurements were carried out between 09:00 and 11:00 h. During the measurements the air relative humidity was about 80%, the leaf temperature ranged from 22 to 24°C and the ambient CO_2_ concentration was about 355 μmol mol^-1^.

### Antioxidant enzyme assay

Plant tissues were **gr**ound in liquid nitrogen and extracted with 2.5 ml extraction buffer containing 50 mM K-phosphate buffer (pH 7.5), 0.1 mM EDTA, 0.3% (w/v) Triton X-100, and 4% (w/v) soluble polyvinylpolypyrrolidone. The extract was then centrifuged at 12,000 × *g* for 30 min at 4°C, and the supernatant was collected for the enzyme activity assay. SOD activity was determined as described previously [38]. Activity of CAT was determined by following the protocol of Deng et al. [39]. Total protein content was measured with the Bradford method [40] using bovine serum albumin (BSA) as a standard.

### Pathogen-related enzyme assay

As for phenylalanine ammonia-lyase (PAL) assay, 100 μl of crude plant extracts were mixed with 900 μl of 6 μM L-phenylalanine and 0.5 M Tris-HCl buffer solution. The mixture was placed in a water bath at 37°C for 70 min. PAL activity was determined as previously described [7]. The amount of trans-cinnamic acid formed from L-phenylalanine was measured at 290 nm using UV-Vis spectroscopy (Shimadzu, Kyoto, Japan). As for polyphenol oxidase (PPO) assay, 200 μl of root extracts used for the PPO (catechol oxidase) assay were mixed with 700 μl of homogenization buffer [8]. The rate of increase in absorbency at λ_420_ was measured for 1 min after the addition of 100 μl 0.2 M catechol (Shimadzu, Kyoto, Japan).

### Data analysis

All data were analyzed using Duncan’s multiple range test (*p* < 0.05) using SPSS 13.0 software (IBM Corp., Armonk, NY, USA). Five replicates were analyzed for each stress treatment.
